# Pharmacological Prevention of Post-Endoscopic Retrograde Cholangiopancreatography Pancreatitis: Where Do We Stand Now?

**DOI:** 10.7759/cureus.10115

**Published:** 2020-08-29

**Authors:** Wiqas Ahmad, Nkechi A Okam, Chenet Torrilus, Dibyata Rana, Mst. Khaleda Khatun, Nusrat Jahan

**Affiliations:** 1 Internal Medicine, California Institute of Behavioral Neurosciences & Psychology, Fairfield, USA

**Keywords:** : post ercp pancreatitis, post ercp pancreatitis and nsaids, pharmacologic prophylaxis in post ercp pancreatitis

## Abstract

Post-endoscopic retrograde cholangiopancreatography pancreatitis (PEP) is the most frequently occurring complication of endoscopic retrograde cholangiopancreatography (ERCP). PEP is associated with significant morbidity and mortality; that is why the prevention of PEP is essential. Pharmacoprevention holds a central position in PEP prophylaxis. The current literature explores the efficacy of various pharmacological agents in preventing PEP, their routes of administration, and the correct administration timing. Data was collected on PubMed using regular keywords, the latter yielded 2077 papers. After applying inclusion and exclusion criteria, 218 papers were selected and screened and 28 studies were finally chosen after the removal of duplicate and irrelevant studies. The selected 28 articles comprised 25 randomized clinical trials and three systematic reviews.

The study concludes that rectal non-steroidal anti-inflammatory drugs (NSAIDs) administered before ERCP are effective in preventing PEP in high-risk patients. The efficacy of rectal NSAIDs in low to medium risk group is not well established. A combination of rectal NSAIDs and intravenous hydration provides improved prophylaxis against PEP in high-risk patients than NSAIDs alone. Nafamostat, sublingual nitrates, and intravenous hydration are potential alternatives in patients with contraindications to NSAIDs.

## Introduction and background

Endoscopic retrograde cholangiopancreatography (ERCP) is a procedure that is used in the diagnosis and management of hepatobiliary and pancreatic disorders [1]. The most frequently occurring complication of ERCP is pancreatitis, and its incidence is approximately 2 to 7% [2]. However, post-endoscopic retrograde cholangiopancreatography pancreatitis (PEP) is severe in 0.1 to 0.5% cases only [3]. PEP has significant financial and social repercussions. The annual expenditure of PEP is estimated to be around 0.20 billion USD and the annual fatality is about 0.7% in the United States of America [3]. PEP has been described as the development of new pancreatic-type epigastric pain associated with at least three times rise in serum amylase/lipase levels occurring 24 hours post ERCP, with pain sufficiently severe to require hospital admission or prolong the stay of an admitted patient [4]. According to the European Society of Gastrointestinal Endoscopy (ESGE), risk factors associated with increased risk of PEP are female gender, suspected sphincter of Oddi dysfunction, prior episode of pancreatitis, prolonged cannulation time, passing guide wire into pancreatic duct more than once and injection of contrast into the pancreatic duct [5].

Different postulated factors may act independently or aggregately to trigger PEP, but the end point of all these mechanisms is to activate inflammatory pathways. Targeting these pathways by preventive treatment options like drugs or addressing various technical issues in ERCP can be used to prevent PEP [6]. Even after improvements in techniques and expertise of endoscopists, the incidence of PEP is still high. Although pancreatic stent placement is an effective way of decreasing the risk of PEP in high-risk patients, it is difficult to perform, and usually, it is done at the end of ERCP. There is no standard preventive strategy to avoid this complication. That is why more than 35 different drugs have been studied in clinical trials until now [7]. Non-steroidal anti-inflammatory drugs (NSAIDs) are the only class of drugs that have been effective in preventing PEP. Though indomethacin administration through the rectal route has been commonly practiced in the past few years, suitable candidates for this drug and appropriate administration timing have not been appropriately established [8].

In this review, we aim to explore pharmacological agents that are effective in the prevention of PEP. Although NSAIDs have shown promising results in various studies, it is still unknown whether all NSAIDs are equally effective and whether or not all patients should receive prophylaxis. Additionally, we endeavor to explore other pharmacological agents besides NSAIDs that are useful in PEP prophylaxis. And finally, we will evaluate whether a combination of NSAIDs with potential pharmacological options offers any additional protection against PEP.

## Review

Literature was searched in PubMed using regular keywords for data collection. Table 1 shows regular keywords and their records.

**Table 1:** Showing search results of regular keywords. ERCP: endoscopic retrograde cholangiopancreatography, NSAIDs: non-steroidal anti-inflammatory drugs

Regular keyword	Total Records Available
Post ERCP Pancreatitis	1810
Post ERCP Pancreatitis and NSAIDs	190
Pharmacologic Prophylaxis in Post ERCP Pancreatitis	77

The following inclusion criteria were used in study selection for this review article: human subjects only, studies related to PEP only, papers published in English language and within the last 10 years, only randomized clinical trials (RCTs) and systematic reviews (SR), all studies available in full-text form.

The following exclusion criteria were used: animal studies, studies published in languages other than English, observational studies, case reports, case series, meta-analysis, non-RCT trials.

Table 2 summarizes results after applying inclusion and exclusion criteria for the regular keyword “Post ERCP Pancreatitis.”

**Table 2:** Applying inclusion /exclusion criteria for selection of studies. RCT: randomized clinical trial, ERCP: endoscopic retrograde cholangiopancreatography

Regular keyword- Post ERCP Pancreatitis
Total Records 1810
Inclusion /Exclusion
Humans 1467
English Language 1352
Published within the last ten years 792
RCTs and Systematic Reviews 161
Full Text 158

Table 3 shows results after applying the inclusion/exclusion criteria for the regular keyword “Post ERCP Pancreatitis and NSAIDs.”

**Table 3:** Applying inclusion/exclusion criteria for selection of studies. RCT: randomized clinical trial, ERCP: endoscopic retrograde cholangiopancreatography, NSAIDs: non-steroidal anti-inflammatory drugs

Regular keyword- Post ERCP Pancreatitis and NSAIDs
Total Records 190
Inclusion /Exclusion
Humans 160
English Language 154
Published within the last ten years 135
RCTs and Systematic Reviews 48
Full text 48

Table 4 shows results after applying the inclusion/exclusion criteria for the regular keyword “Pharmacologic prophylaxis in Post ERCP pancreatitis.”

**Table 4:** Applying inclusion/exclusion criteria for selection of studies. RCT: randomized clinical trial, ERCP: endoscopic retrograde cholangiopancreatography

Regular keyword- Pharmacologic prophylaxis in Post ERCP Pancreatitis
Total Records 77
Inclusion /Exclusion
Humans 64
English Language 63
RCTs and Systematic Reviews 12
Published within the last 10 years 34
Full Text 12

**Figure 1:**
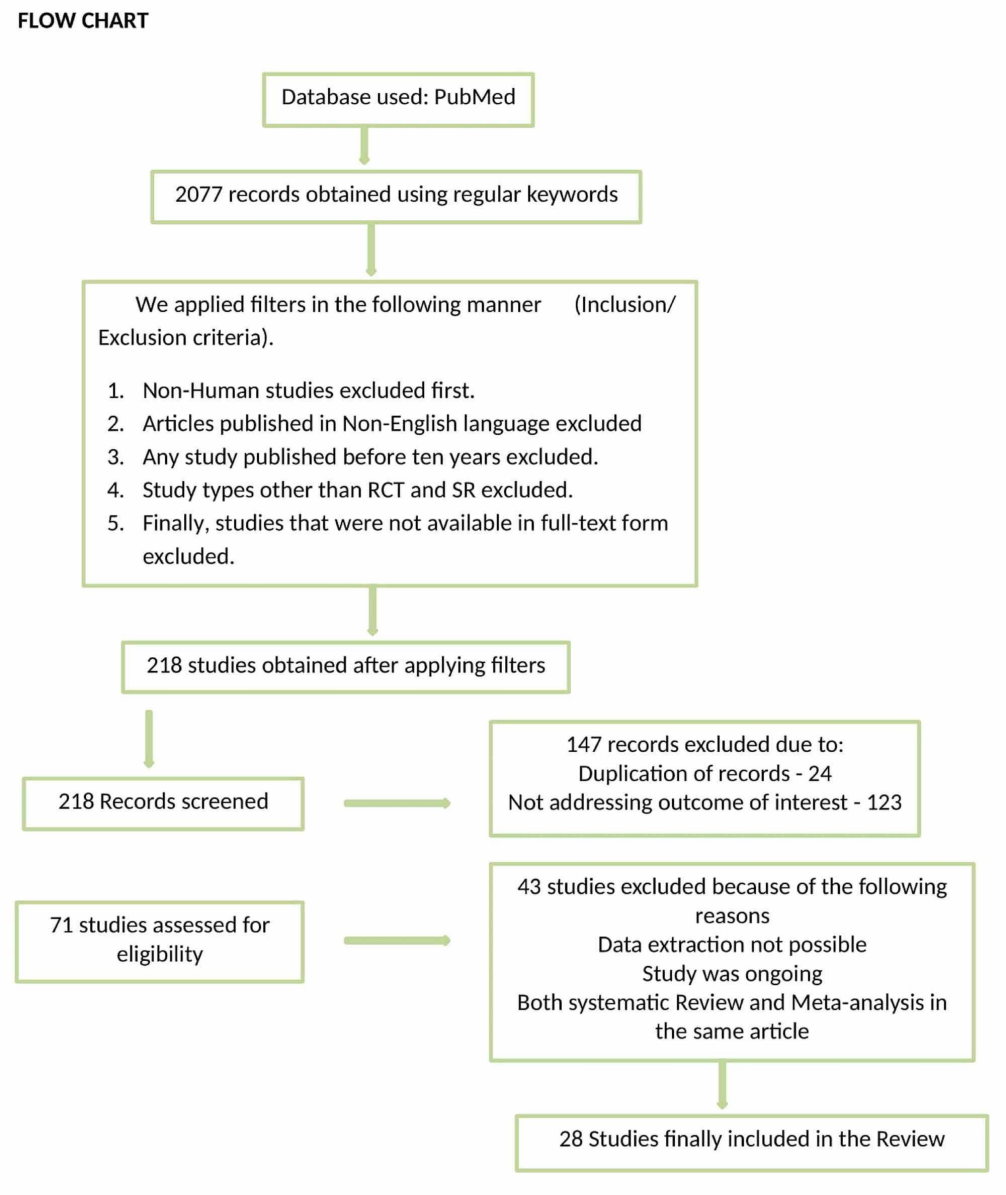
Showing selection process of studies for this review. RCT: randomized clinical trial, SR: systematic review

A total of 147 articles were excluded from the study either because they were duplicate studies or did not address the outcome of interest (pharmacological prevention in PEP). After a refined search, 71 articles were considered eligible for inclusion. We reviewed all 71 studies, and excluded 43 studies either because data extraction was not possible, studies were still ongoing and results were not available, or they included both systematic review and meta-analysis in one study. Finally, 28 studies were included, which comprised 25 RCTs and three systematic reviews. Figure 1 shows the selection process of studies.

Discussion

Pharmacological prophylaxis is a crucial component of post-ERCP pancreatitis (PEP) prevention, and a significant number of pharmacological agents have been investigated in PEP prophylaxis. The literature review would explore the efficacy of different pharmacological agents in the prevention of PEP, their route of administration, and the correct timing of administration.

Non-Steroidal Anti-Inflammatory Drugs (NSAIDs)

Non-steroidal anti-inflammatory drugs (NSAIDs) are the only class of drugs that has shown significant benefit in PEP prophylaxis. In the current literature review, eight studies explored NSAIDs' role in PEP prevention. Table 5 summarizes the main results of these studies.

**Table 5:** Summarizing results of studies. ERCP: endoscopic retrograde cholangiopancreatography,  PEP: post endoscopic retrograde cholangiopancreatography pancreatitis,  NSAIDs: non-steroidal anti-inflammatory drugs.

Author/ year	Sample Size	Drug Used	Route and Timing Of Drug Administration	Main Points	P -value
Andrade-Davila et al.,2015 [7].	166	indomethacin	Rectal (administered after ERCP)	Study was conducted in patients at high risk of PEP.PEP developed in 4.87 % (indomethacin group) vs. 20.23 % (Placebo group) . Rectal indomethacin effectively decreased risk of PEP.	P=0.01
Elmunzer et al. ,2012 [9].	602	indomethacin	Rectal (administered after ERCP)	PEP developed in 9.2% (indomethacin group) vs. 16.9 % (placebo group).Rectal indomethacin Significantly reduced PEP development in patients at high risk of developing PEP.	P=0.005
Mansour Ghanaei et al. ,2016 [10].	324	naproxen	Rectal (Before ERCP)	PEP occurred more commonly in the Placebo group than the naproxen group.	P<0.01
Otsuka T et al. ,2012 [11].	104	diclofenac	Rectal (Before ERCP)	PEP developed in 3.9 %( diclofenac group) vs. 18.9 %( control group). The study showed that a low dose of the diclofenac effectively reduced the risk of PEP.	P=0.017
Kubilian NM et al. ,2015 [12].	N/A	NSAIDs	Rectal (Before/After ERCP)	This systematic review concluded that rectal NSAIDs reduce the risk of PEP in the high risk group.	
Levenick et al. ,2016 [13].	449	indomethacin	Rectal (during procedure drug was given)	The study assessed the prophylactic benefit of indomethacin in all patients irrespective of their risk of developing PEP (around 2/3 of the patents were at average risk of PEP.)Incidence of PEP in indomethacin group (7.2 %) and placebo group (4.9 %) indicated that indomethacin in this study did not prevent risk of developing PEP.	P=0.33
Dobronte Z et al. , 2014 [14].	686	indomethacin	Rectal (Before ERCP)	Incidence of PEP in the control group (6.9%) was not significantly different from the indomethacin group (5.8%). In this study indomethacin did not prove effective in PEP prevention.	P=0.54
Mohammad Alizadeh et al. ,2017 [15]	372	diclofenac/ indomethacin/naproxen	Rectal (Before ERCP)	Incidence rate of PEP was 2.4 % in the diclofenac group,3.4 % in the indomethacin group and 10.3 % in naproxen group. This study showed that diclofenac and indomethacin are more effective in reducing PEP	P =0.001

Many of the RCTs and the systematic analysis we reviewed in this study have shown that NSAIDs are effective in reducing the risk of PEP [7,9-12], although a few RCTs have shown that NSAIDs are not useful in PEP prevention [1,13,14]. NSAIDs provide improved prophylaxis in patients at high risk of developing PEP, as demonstrated by multiple studies [7,9]. The study conducted by Levenick et al. in patients at average risk of ERCP showed that rectal NSAIDs may not have prophylactic benefits in average-risk patients [13]. Among NSAIDs, diclofenac and indomethacin are more effective than naproxen in PEP prophylaxis [15]. The administration route is also significant as NSAIDs administered via the rectal route effectively prevents PEP [7,9,11,12]. In contrast, the administration of NSAIDs through other routes has not been found useful [1,16,17]. There is still some controversy regarding the appropriate timing of NSAIDs administration (before/during/after ERCP). However, the analysis of most of the studies in our review showed that NSAIDs administration before ERCP is the most suitable option. The recommendation from the European Society for Gastrointestinal Endoscopy (ESGE) is that all patients undergoing ERCP should receive prophylactic rectal indomethacin or diclofenac provided they have no contraindications to NSAIDs [5].

NSAIDs are cheap, readily available, and highly effective in PEP prevention; therefore, they should be used in all patients at high risk of developing PEP, provided they do not have any conditions that may preclude its use. Rectal NSAIDs, in conjunction with intravenous hydration, are another viable alternative that can be used in high-risk patients. There is little evidence to support the use of prophylactic rectal NSAIDs in low to medium risk patients. Further studies in this regard may be a possible area of research for the future.

Intravenous Hydration

Intravenous hydration can prevent PEP by maintaining sufficient perfusion to the pancreas and thereby suppressing the inflammatory cascade within the pancreas [18]. Recent evidence indicates that hydration with Ringer's lactate (RL) solution is better than normal saline because RL solution decreases the risk of developing systemic inflammatory response more than normal saline [19].

A systematic review performed by Smeets et al. evaluated the role of hydration in PEP prophylaxis and revealed that intravenous hydration offers some protection against PEP. However, the evidence was not entirely conclusive [18]. In 2018, Park et al. conducted an RCT, the results of the study showed that aggressive hydration with Ringer's lactate prevents PEP occurrence in patients with average to high risk [20].

Intravenous hydration is a potential option for patients at high risk of developing PEP, particularly if they also have contraindications to NSAIDs.

Combination of Rectal NSAIDs and Intravenous Hydration

Though rectal NSAIDs alone have shown encouraging results in PEP prophylaxis but still PEP occurs despite NSAIDs usage in high-risk patients. A study conducted by Hosseini et al. has demonstrated that a combination of rectal indomethacin with intravenous normal saline significantly reduced the incidence of PEP [21]. Another study conducted by Mok et al. revealed that the combination of Ringer’s lactate and rectal indomethacin was also quite effective in preventing PEP in patients at high risk of PEP [22].

Therefore, it seems reasonable that in patients at high risk of PEP, both rectal NSAIDs and intravenous hydration are used.

Somatostatin

Somatostatin acts by inhibiting secretions from the pancreas and its role in preventing PEP has been explored in various studies. A study conducted by Bai et al. suggested that somatostatin effectively reduces amylase levels and prevents PEP [23], while a systematic review revealed that somatostatin has moderate benefit in PEP prophylaxis [12]. Two other studies showed that somatostatin is not useful in preventing PEP [24,25].

Currently, somatostatin is not recommended for PEP prevention because there is little evidence that it is useful in reducing the risk of PEP.

Antioxidants

A systematic review was conducted to determine the role of antioxidants such as allopurinol, beta carotene, selenite, pentoxifylline, and N-acetyl cysteine in the prevention PEP and concluded that antioxidants are not useful in PEP prophylaxis [2].

Protease Inhibitors

Protease inhibitors have been considered a possible treatment option in acute pancreatitis due to these drugs' ability to block different enzymes and inflammatory cascade in the pancreas [26]. Various protease inhibitors like nafamostat and ulinastatin have also been investigated in the prevention of post-ERCP pancreatitis.

Nafamostat has been found effective in reducing the incidence of PEP [26]. Another study done by Park and his team showed that both nafamostat and ulinastatin are equally useful in PEP prophylaxis [27]. Kubilian and his team also found in their systematic review that nafamostat effectively prevents PEP [12].

Though nafamostat appears promising in PEP prophylaxis, it has not been widely used because it is quite expensive and needs to be administered through the intravenous route.

Nitrates

Nitroglycerin is a drug that reduces the sphincter of Oddi pressure by causing smooth muscle relaxation, and it also enhances blood flow to the pancreas [12].

Katsinelos et al. found that administering a combination of nitroglycerin and glucagon makes it more likely to selectively cannulate common bile duct and reduce PEP incidence [28].

A systemic review performed by Kubilian and his team showed mixed results; they analyzed seven studies of which three RCTs showed a benefit of nitroglycerin in decreasing PEP while four RCTs did not demonstrate any benefit. The two studies that used sublingual nitrates revealed that it is effective in reducing PEP [12].

The evidence for nitrate efficacy is modest and is not recommended for PEP prophylaxis. However, sublingual nitrates offer a potential prophylactic option in those with contraindications to NSAIDs. Further studies in the future will help define the role of sublingual nitrates in PEP prophylaxis clearly.

Limitations

The current literature review has few limitations. Only randomized clinical trials and systematic reviews conducted after 2010 were included. Many studies assessed the function of pharmacoprevention in high-risk patients developing PEP so that potential research may be done to investigate the role of pharmacoprevention in low to medium risk groups.

## Conclusions

Pharmacoprevention is an effective method for the prevention of PEP. The review concludes that rectal NSAIDs administered before ERCP are successful in preventing PEP in high-risk populations. The use of NSAIDs by other routes is not effective in PEP prophylaxis. The effectiveness of NSAIDs in patients with low to medium risk is not well established, and this could be a possible area for further investigations. Intravenous hydration with lactated ringer solution provides some prophylaxis, but a combination of rectal NSAIDs and intravenous hydration is a more suitable option for high-risk patients. Possible options are available in patients with contraindications to NSAIDs such as intravenous hydration, nafamostat and sublingual nitrates, but further studies in the future are needed to elucidate the role of these drugs in PEP prophylaxis.
